# Prevalence of transthyretin amyloid cardiomyopathy in an unselected cohort with heart failure with preserved ejection fraction

**DOI:** 10.1007/s12471-025-01954-3

**Published:** 2025-04-28

**Authors:** Anouk Achten, Vanessa P. M. van Empel, Jerremy Weerts, Sanne Mourmans, Hans-Peter Brunner-La Rocca, Sandra Sanders-van Wijk, Christian Knackstedt

**Affiliations:** 1 Department of Cardiology, Cardiovascular Research Institute Maastricht (CARIM), Maastricht University Medical Centre (MUMC+), Maastricht, The Netherlands; 2 Department of Cardiology, Zuyderland Medical Centre, Heerlen, The Netherlands

**Keywords:** Amyloid cardiomyopathy, Transthyretin amyloidosis, Heart failure with preserved ejection fraction, Prevalence, Left ventricular wall thickness

## Abstract

**Introduction:**

Heart failure with preserved ejection fraction (HFpEF) represents a heterogeneous syndrome characterised by various underlying aetiologies, such as transthyretin amyloid cardiomyopathy (ATTR-CM). The aim of this study was to determine the true prevalence of ATTR-CM in a Dutch all-comers cohort of HFpEF patients.

**Methods:**

From 2018 to 2023, all patients diagnosed with HFpEF underwent prospective screening for ATTR-CM. Diagnosis of ATTR-CM was made in accordance with guideline recommendations.

**Results:**

Of the 202 HFpEF patients included (mean ± standard deviation age: 76 ± 7 years; 64% female), 9 (5%) showed cardiac uptake on scintigraphy, of whom 6 (3%) were subsequently diagnosed with wild-type ATTR-CM. Left ventricular wall thickness (LVWT) was significantly higher in ATTR-CM patients than non-amyloid HFpEF patients (median interventricular septum diameter: 15 mm; interquartile range (IQR): 11–17 vs 10 mm; IQR: 9–11; *p* < 0.001). Interestingly, 2 ATTR-CM patients (33%) did not have increased LVWT at the time of diagnosis. These 2 patients were in a less advanced prognostic stage.

***Conclusion*:**

This study revealed a low prevalence of ATTR-CM (3%) in an unselected HFpEF cohort. We identified ATTR-CM patients without increased LVWT (33%), who presented at an earlier disease stage. Hence, relying exclusively on LVWT for the diagnosis of ATTR-CM may result in delayed and/or missed diagnoses.

## What’s new?

- To our knowledge, this is the first study that screened an unselected cohort of patients with heart failure with preserved ejection fraction (HFpEF) to determine the true prevalence of transthyretin amyloid cardiomyopathy (ATTR-CM).
- Previous studies exclusively included HFpEF patients with an increased left ventricular wall thickness, potentially overlooking those in an early disease stage without such left ventricular hypertrophy.
- Early diagnosis of ATTR-CM is imperative as current treatments only mitigate amyloid formation and disease progression rather than achieve complete cessation.

## Introduction

Heart failure with preserved ejection fraction (HFpEF) represents a heterogeneous syndrome. Transthyretin amyloid cardiomyopathy (ATTR-CM) has been identified as a cause of HFpEF, with reported prevalences rates ranging from 5 to 25% [1–6]. Diagnosing ATTR-CM early is imperative, as current treatments only mitigate amyloid formation and disease progression rather than achieve complete cessation [7].

Currently, ATTR-CM is suspected in HFpEF patients when left ventricular wall thickness (LVWT) ≥ 12 mm [7]. However, recent studies have indicated that manifestations of amyloid deposition appear years before ATTR-CM is diagnosed [8, 9]. These early manifestations highlight the fact that ATTR-CM is a slowly progressive disease, with increased LVWT appearing at a later disease stage [10]. Detection of ATTR-CM prior to the onset of LV hypertrophy could provide an opportunity for therapeutic interventions to halt disease progression in its early stages by preventing further amyloid deposition.

**Fig. 1:**
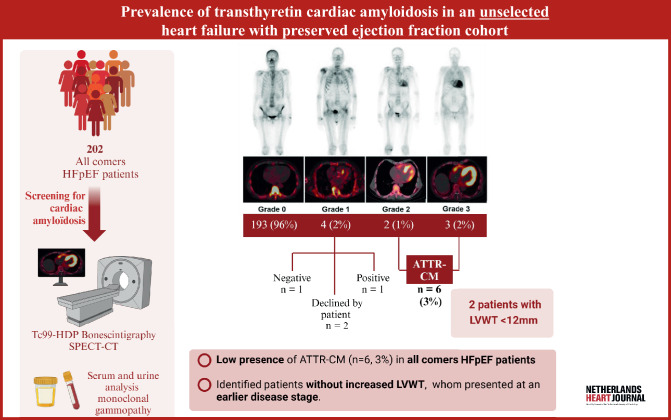
Infographic of prevalence of transthyretin cardiac amyloidosis in an unselected heart failure with preserved ejection fraction cohort. (Created with BioRender)

Previous studies have predominantly included HFpEF patients with increased LVWT [2, 3], potentially overlooking those in an early disease stage without such LV hypertrophy [10]. To determine the true prevalence of ATTR-CM, we systematically screened an all-comers HFpEF population for ATTR-CM, irrespective of LVWT (Fig. 1).

## Methods

### Study population

From 2018 to 2023, all HFpEF patients attending a specialised HFpEF outpatient clinic were prospectively screened for ATTR-CM. This HFpEF outpatient clinic was set up to systematically diagnose HFpEF in patients referred for symptoms of this disease. Most patients referred to the clinic had previously undergone echocardiography demonstrating a preserved LV ejection fraction (LVEF), raising suspicion of HFpEF. Only patients subsequently diagnosed with HFpEF were included in this study. HFpEF diagnosis was based on the 2016 European Society of Cardiology definition. Exclusion criteria included prior evaluation for amyloid cardiomyopathy, LVEF < 50% or unwillingness to participate. Written informed consent was obtained from all included patients, and the local medical ethics review committee approved the study protocol (nr. NL76585.068.21).

### Screening for transthyretin amyloid cardiomyopathy

HFpEF patients were screened for ATTR-CM using bone scintigraphy with single-photon emission computerized tomography, as well as serum and urine analysis for protein electrophoresis, immunofixation and light chain quantification [7]. ATTR-CM diagnosis was based on positive bone scintigraphy (^99m^Tc–hydroxymethylene diphosphonate; Perugini grade II or III) with the absence of monoclonal gammopathy. In inconclusive cases (Perugini grade I), diagnosis relied on a positive cardiac biopsy for transthyretin amyloid, in accordance with current guidelines [7].

Information on the presence of typical cardiac and extracardiac red flags was extracted from medical records, including LVWT ≥ 12 mm [6], atrial fibrillation [11], aortic valve sclerosis with restriction [12], unilateral or bilateral carpal tunnel syndrome [13], trigger finger [14], polyneuropathy [15], spinal canal stenosis [16], hip or knee arthroplasty [17], presence of a pacemaker [18], conduction delay (PQ duration ≥ 200 ms or QRS duration ≥ 120 ms) or micro-voltage signals on electrocardiography [7].

### Statistical analysis

Descriptive analyses were conducted to examine and compare demographics, comorbidities, red flags for ATTR-CM, biological data and echocardiographic measurements between patients diagnosed with ATTR-CM and non-amyloid HFpEF patients. Categorical and ordinal variables are presented as number (percentage) and were compared using the Fisher’s exact test or Mann-Whitney U test, as appropriate. Continuous variables are expressed as mean ± standard deviation (SD) or median and interquartile range (IQR) and were compared using the Mann-Whitney U test when applicable. All statistical analyses were performed using RStudio version 2021.09.01 (Boston, MA, USA). A *p*-value of < 0.05 was considered statistically significant.

## Results

**Fig. 2:**
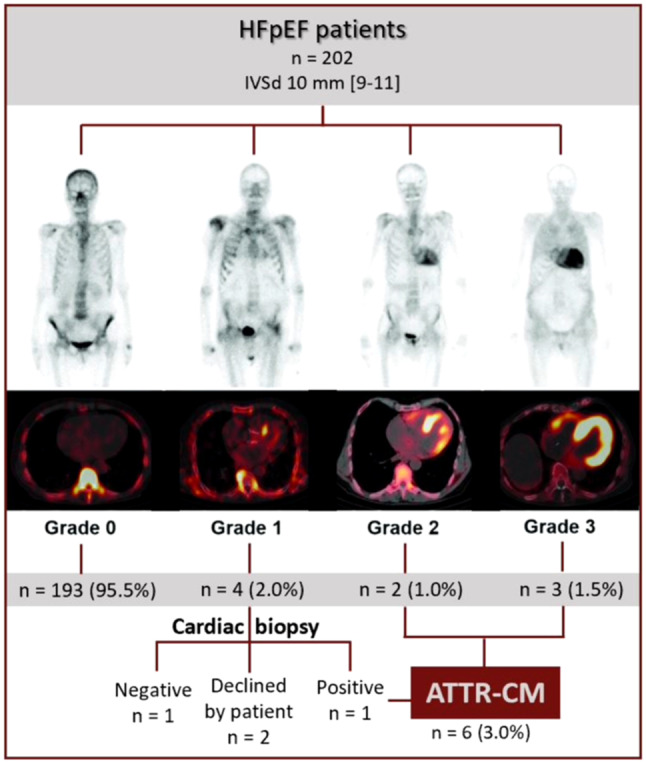
Prevalence of transthyretin amyloid cardiomyopathy (*ATTR-CM*) in patients with heart failure with preserved ejection fraction (*HFpEF*). *IVSd* interventricular septum diameter

This study included 202 consecutive HFpEF patients (mean ± SD age: 76 ± 7 years; 64% female) (Tab. 1). A total of 9 HFpEF patients (5%) showed cardiac tracer uptake on bone scintigraphy (Perugini grade ≥ I) (Fig. 2). Bone scintigraphy was inconclusive in 4 patients, 2 of whom underwent subsequent endomyocardial biopsy. The other 2 patients chose not to pursue further investigations and were excluded from further analyses. Endomyocardial biopsy confirmed the presence of ATTR-CM in 1 patient, ultimately resulting in a diagnosis of ATTR-CM in 6 patients (3%) and an inconclusive diagnosis in 2 patients. No TTR gene mutations were found among the patients diagnosed with ATTR-CM. Monoclonal gammopathy was present in 1 ATTR-CM patient, who was subsequently diagnosed with monoclonal gammopathy of unknown significance by a haematologist, without suspicion of light chain amyloidosis.

**Table 1:** Baseline characteristics

Variable	Study population (*N* = 202)	Positive scintigraphy (*n* = 9)	Non-amyloid HFpEF (*n* = 194)	ATTR-CM (*n* = 6)	*P*-value
Female	129 (64%)	2 (22%)	127 (66%)	1 (17%)	** 0.024**
Age (years)	76 ± 7	79 [77–83]	77 [72–81]	79 [78–82]	0.226
*Medical history*					
– Carpal tunnel syndrome	24 (12%)	5 (56%)	19 (10%)	4 (67%)	** 0.002**
– Polyneuropathy	22 (11%)	2 (22%)	20 (10%)	2 (33%)	0.129
– Spinal canal stenosis	22 (11%)	1 (11%)	21 (11%)	1 (17%)	0.510
– Atrial fibrillation/flutter	86 (43%)	7 (78%)	79 (41%)	5 (83%)	0.212
– Pacemaker	24 (12%)	1 (11%)	24 (12%)	0 (0%)	0.129
– Hypertension	159 (78%)	7 (78%)	153 (79%)	4 (67%)	0.604
– Significant CAD	38 (19%)	4 (44%)	35 (18%)	3 (50%)	0.081
– TIA/CVA	31 (15%)	2 (22%)	30 (16%)	0 (0%)	0.593
– Hip/knee arthroplasty	18 (9%)	2 (22%)	17 (9%)	0 (0%)	1.000
– Trigger finger	7 (4%)	1 (11%)	6 (3%)	0 (0%)	1.000
*NYHA class*					0.955
– Class I	3 (2%)	0 (0%)	3 (2%)	0 (0%)	
– Class II	92 (46%)	4 (44%)	89 (46%)	3 (50%)	
– Class III	98 (49%)	5 (56%)	93 (48%)	3 (50%)	
– Class IV	4 (2%)	0 (0%)	4 (2%)	0 (0%)	
*Echocardiography*					
– LVEF (%)	60 [56–64]	60 [56–61]	60 [57–64]	58 [54–62]	0.469
– LVEDD (mm)	48 [44–51]	44 [40–51]	48 [44–51]	43 [39–50]	0.094
– LVIVSd (mm)	10 [9–11]	12 [11–15]	10 [9–11]	15 [11–17]	**<** **0.001**
– LVPWd (mm)	9 [8–10]	11 [10–14]	9 [8–10]	12 [10–15]	** 0.002**
– LVMI (g/m^2^)	81 [69–96]	106 [90–107]	80 [69–94]	104 [83–164]	** 0.037**
– LAVI (ml/m^2^)	45 [36–55]	54 [49–72]	44 [34–55]	53 [39–76]	0.176
– e’ septal (cm/s)	6.3 [5.1–7.8]	5.3 [4.8–5.8]	6.5 [5.1–7.8]	5.4 [4.7–10.6]	0.645
– e’ lateral (cm/s)	8.6 [6.6–10.5]	7.5 [5.9–8.4]	8.6 [6.6–10.7]	7.8 [6.4–8.6]	0.442
– E/e’ average	11.2 [9.2–14.7]	12.6 [11.5–13.6]	11.2 [9.1–14.9]	11.9 [10.7–13.6]	0.659
– Aortic valve stenosis	15 (7%)	0 (0%)	15 (8%)	0 (0%)	1.000
*Electrocardiography*					
– PQ duration (ms)	172 [148–192]	177 [177–178]	172 [148–192]	177 [176–178]	0.610
– QRS duration (ms)	92 [84–110]	88 [84–148]	96 [84–113]	88 [81–156]	0.736
– Conduction delay	65 (32%)	3 (33%)	63 (33%)	2 (33%)	1.000
– Micro-voltage signals	22 (11%)	2 (22%)	20 (10%)	2 (33%)	0.145
*Laboratory testing*					
– eGFR (ml/min per 1.73 m^2^)	59 [40–73]	45 [39–64]	58.8 [40.7–72.7]	48.4 [39.4–80.6]	0.902
– NT-proBNP (pg/ml)	546 [287–1199]	1362 [896–3154]	541 [271–1180]	2258 [787–6410]	** 0.011**
– hsTnT (ng/l)	19 [14–36]	47 [27–123]	17 [13–35]	47 [13–252]	0.254

Data are presented as *n* (%), mean ± standard deviation or median [interquartile range]

*HFpEF* heart failure with preserved ejection fraction, *ATTR-CM* transthyretin amyloid cardiomyopathy, *CAD* coronary artery disease, *TIA* transient ischaemic attack, *CVA* cerebrovascular accident, *NYHA* New York Heart Association, *LVEF* left ventricular ejection fraction, *LVEDD* left ventricular enddiastolic diameter, *LVIVSd* left ventricular interventricular septum diameter, *LVPWd* left ventricular posterior wall diameter, *LVMI* left ventricular mass index, *LAVI* left atrial volume index, *eGFR* estimated glomerular filtration rate, *NT-proBNP* N-terminal pro B–type natriuretic peptide, *hsTnT* high sensitive troponin T

Of the 6 patients diagnosed with ATTR-CM, all but 1 were male, resulting in a significantly higher proportion of male patients compared with non-amyloid HFpEF patients (83% vs 35%; *p* = 0.024). Furthermore, echocardiographic LVWT was significantly higher in patients with ATTR-CM compared with non-amyloid HFpEF patients, with a median interventricular septum diameter of 15 mm (IQR: 11–17) and 10 mm (IQR: 9–11), respectively (*p* < 0.001), and a median posterior wall diameter of 12 mm (IQR: 10–15) and 9 mm (IQR: 8–10), respectively (*p* = 0.002) (Tab. 1). Two ATTR-CM patients (33%; 95% confidence interval: 4–78%) did not have increased LVWT (≥ 12 mm). These 2 patients were in the less advanced National Amyloidosis Centre (NAC) prognostic stage I [19]. In contrast, 3 out of the 4 ATTR-CM patients with increased LVWT were diagnosed with the more advanced prognostic stages II and III. Moreover, the 2 patients were classified as New York Heart Association (NYHA) class II, whereas all but 1 of the patients with increased LVWT were classified as NYHA class III. Nonetheless, both ATTR-CM patients without increased LVWT exhibited other typical red flags indicative of transthyretin amyloidosis.

Considering typical red flags indicative of ATTR-CM, 81% of the entire HFpEF patient cohort showed ≥ 1 identifiable red flags. Even when atrial fibrillation—commonly observed in HFpEF—was excluded as a red flag, 142 patients of the entire cohort (71%) still presented with ≥ 1 red flags for ATTR-CM. Carpal tunnel syndrome was significantly more prevalent in ATTR-CM patients than non-amyloid HFpEF patients (67% vs 10%; *p* = 0.002), and ATTR-CM patients had a higher median number of typical red flags (3; IQR: 2–4 vs 1; IQR: 1–2; *p* = 0.001). The frequencies of other specific red flags or comorbidities did not differ significantly (Tab. 1).

## Discussion

Current guidelines recommend active screening for ATTR-CM in HFpEF patients with unexplained LVWT ≥ 12 mm and ≥ 1 typical red flags for amyloid cardiomyopathy [7]. In light of this directive, our study has several noteworthy findings.

First, we found an ATTR-CM prevalence of 3% in an unselected HFpEF cohort, which is lower than that previously reported [1–3, 20]. This can be attributed to the fact that previous studies primarily included HFpEF patients with LVWT ≥ 12 mm [1–3], thereby selecting a subgroup with a higher ATTR-CM prevalence. The study by Healy et al. is one of only 2 studies that included HFpEF patients without explicitly increased wall thickness [20]. However, even in their cohort, 62% of the patients had LVWT ≥ 12 mm (median: 12 mm). The other study, by Devesa et al., included hospitalised HFpEF patients with LVWT < 12 mm and reported an ATTR-CM prevalence of 5% [5]. In our outpatient clinic, we included both HFpEF patients with and without LV hypertrophy but found an even lower ATTR-CM prevalence. This may be partly explained by the fact that 64% of our patients were female, whereas ATTR-CM is known to be more common in men [21]. Initially, there was concern that female patients with ATTR-CM are underdiagnosed [21], but our findings do not support this hypothesis. Despite screening a large proportion of women, we identified very few cases of ATTR-CM. The high representation of female patients in our HFpEF cohort may therefore contribute to the low ATTR-CM prevalence observed.

Second, we identified 2 ATTR-CM patients (33%) without increased LVWT, who were in an early disease stage, as shown by their NAC prognostic stage and NYHA class. This supports the concept that cardiac uptake on bone scintigraphy serves as an indicator of ATTR-CM presence rather than amyloid fibril quantity and may thus identify ATTR-CM before clinically evident LV hypertrophy [5, 10]. If we had followed the current guidelines, these patients would have been overlooked or diagnosed at a later disease stage [7]. Consequently, to facilitate early ATTR-CM diagnosis, it may be necessary to broaden the scope beyond patients with increased LVWT and consider other patient groups as well.

Currently, there is a strong focus on using HFpEF patients as a screening group for ATTR-CM [7]. However, it is well established that over half of the ATTR-CM patients have a reduced or mildly reduced LVEF [22]. This underscores that ATTR-CM can manifest across various cardiology subgroups, highlighting the urgent need for diagnostic indicators beyond echocardiography.

Last, we observed a high prevalence of typical red flags for ATTR-CM in all HFpEF patients. Specifically, 81% of all HFpEF patients exhibited ≥ 1 red flags indicative of ATTR-CM. These findings highlight the diagnostic challenge for ATTR-CM, particularly in early disease stages. Routine bone scintigraphy in an unselected HFpEF population remains controversial due to the low prevalence of ATTR-CM in this group and the significant number of scans required. Therefore, we do not recommend standardised screening for ATTR-CM in all HFpEF patients but emphasise the urgent need for more specific ATTR-CM indicators beyond the currently described red flags and echocardiographic parameters. Meanwhile, targeted screening of HFpEF patients using existing screening tools may be a practical approach in clinical practice [13], as screening remains essential, particularly with the increasing number of treatment options available, even for patients in advanced disease stages [23].

### Study limitations

Our results may be affected by selection bias as the entire patient cohort was predominantly referred by cardiologists, with only a small proportion referred by general practitioners. However, our hospital functions as both a regional and university hospital, catering to a diverse patient population representative of the local community. We believe our cohort broadly reflects the HFpEF population, as all patients suspected of HFpEF are evaluated systematically, irrespective of LVWT or ATTR-CM–related red flags. Nonetheless, we acknowledge that the specialised nature of our HFpEF clinic and the diagnostic expertise of referring cardiologists may introduce a degree of pre-selection bias, potentially impacting the ATTR-CM prevalence. Moreover, the high proportion of female patients in our cohort may have contributed to the low prevalence of ATTR-CM observed.

Furthermore, the decision of 2 patients to decline further evaluation for ATTR-CM following inconclusive bone scintigraphy may have influenced the ATTR-CM prevalence in our cohort, particularly given its already low rate. Nonetheless, we believe that the clinical relevance of these 2 cases is limited considering the overall very low prevalence of ATTR-CM in an unselected HFpEF cohort. Therefore, we excluded these patients from further analyses.

## Conclusion

This investigation revealed a low prevalence of ATTR-CM (3%) in unselected cohort of 202 HFpEF patients. We identified 2 ATTR-CM patients (33%) without increased LVWT, who presented at an earlier disease stage. Hence, relying solely on LVWT for ATTR-CM diagnosis may lead to delayed and/or missed diagnoses. Furthermore, 71% of all HFpEF patients had ≥ 1 red flags indicative of ATTR-CM, highlighting the need for more specific ATTR-CM indicators in this patient population.
